# Utilization of Tubular Retractors for Paramedian Approach in Dorsal Column Spinal Stimulator Paddle Lead Placement: A Technical Report and Literature Review

**DOI:** 10.7759/cureus.86655

**Published:** 2025-06-24

**Authors:** Brandon M Edelbach, Jeffrey Lubisich, Vadim Gospodarev, Rasha Elbadry, Namath Hussain

**Affiliations:** 1 Neurological Surgery, Loma Linda University School of Medicine, Loma Linda, USA; 2 Neurological Surgery, Loma Linda University Medical Center, Loma Linda, USA

**Keywords:** paddle leads, paramedian approach, refractory neuropathic pain, spinal cord stimulation (scs), tubular retractors

## Abstract

Spinal cord stimulation (SCS) is an established therapy for refractory back and leg pain, traditionally utilizing laminectomy-placed paddle leads or percutaneous cylindrical leads. Although percutaneous systems reduce soft tissue injury, they suffer higher migration rates and less precise dorsal column targeting. Minimally invasive surgical (MIS) techniques using tubular retractors combine the stability of paddle electrodes with reduced surgical trauma. Here, we describe a paramedian METRx tubular technique designed to minimize disruption of the posterior tension band while preserving paddle lead advantages. From January 2024 to April 2025, the senior author implanted paddle leads in 130 patients undergoing an SCS trial. Six patients (five men and one woman; mean age 67.7±6.4 years; mean BMI 34.9±4.7 kg/m²) underwent definitive paddle lead placement via a 3 cm paramedian incision and sequential dilation to a 22 mm METRx working channel. Neurosensory monitoring (somatosensory evoked potential (SSEP)/motor evoked potential (MEP)) was maintained throughout. Docking was performed at T7-T11 (four at T8-T9, one at T7-T8, and one at T10-T11). Under direct microscopic/endoscopic visualization, hemilaminectomy and flavectomy expose the dorsal epidural space. Boston Scientific paddle leads (Marlborough, Massachusetts, United States) were fluoroscopically confirmed, anchored to the interspinous ligament, tunneled subfascially to the gluteal pocket, and connected to an implantable pulse generator. All six paddle leads were successfully positioned without intraoperative complications. The mean estimated blood loss was 25 mL, and the mean operative time was 56.0±10.6 minutes. At the first postoperative follow-up (mean 2.9±3.1 months), no lead migrations, revisions, or infections were observed. The average Visual Analog Scale (VAS) pain score improved to 4.5±1.3. Importantly, with the paramedian approach, the midline supraspinous and interspinous ligaments were preserved, thereby reducing the osteoligamentous insult compared to midline or open microdissection techniques. The paramedian METRx tubular technique for paddle lead SCS placement is safe, efficient, and reproducible, with minimal blood loss and operative time. By sparing key posterior tension band structures, it may enhance postoperative recovery and preserve spinal stability. Prospective, controlled studies comparing paramedian versus midline MIS approaches are warranted to further elucidate clinical benefits and long-term outcomes.

## Introduction

Early descriptions of the clinical utilization of spinal cord stimulation (SCS) were reported in 1967 by Shealy and colleagues, who first demonstrated that electrical stimulation of the dorsal spinal columns could effectively modulate chronic pain by placing an electrode directly over the spinal cord [1]. This pioneering work laid the foundation for our current understanding of SCS, which is thought to achieve neuromodulation based on the principles of gate control theory. Specifically, electrodes placed on the dorsal column are believed to preferentially activate large-diameter, non-nociceptive Aβ fibers. This activation engages inhibitory interneurons in the dorsal horn, which suppress the transmission of pain signals carried by small-diameter C fibers, thereby reducing the perception of chronic pain [2].

These initial SCS systems delivered tonic stimulation via surgically implanted paddle electrodes with typically 2-4 electrode contacts in unipolar configurations [3,4]. These paddle leads required a laminectomy for placement. However, in the 1980s and 1990s, percutaneous cylindrical leads were introduced [5,6]. This enabled epidural SCS implantation via a Tuohy needle [7]. These cylindrical leads offered circumferential current delivery via 4-8 ring electrodes [7-9]. The benefits of the minimally invasive percutaneous placement of cylindrical leads are offset by higher rates of migration and less selective dorsal column targeting [10-13].

While the basic design of paddle and cylindrical leads has been preserved in contemporary SCS techniques, significant advancements have occurred in both hardware and stimulation delivery. Modern leads now contain 8-16 contacts, with some systems incorporating sensory electrodes that enable the real-time monitoring of evoked compound action potentials (ECAPs), allowing dynamic adjustment of electrode output [14-16]. Regarding stimulation parameters, the original paradigm of continuous monophasic pulses remains in use. However, the Food and Drug Administration (FDA) has recently approved high-frequency stimulation (10 kHz, compared to the traditional 40-60 Hz) for the treatment of failed back surgery syndrome (FBSS), as it has been shown to modulate pain without producing the paresthesia commonly associated with lower-frequency stimulation [8,9].

These electrode and waveform innovations have transformed SCS into a versatile platform for neuromodulation, enabling tailored therapy for patients with refractory FBSS. In this report, we describe a technique for paddle lead placement via minimally invasive METRx tubular retractors, combining the stability and directional precision of paddle electrodes with the tissue-sparing benefits of a percutaneous approach. While midline minimally invasive surgical (MIS) approaches exist, they disrupt the posterior tension band. This study explores a paramedian alternative to preserve ligamentous integrity.

## Technical report

Patients and methods

Between January 2024 and April 2025, the senior author performed percutaneous lead placement for a therapeutic trial of SCS in 130 patients presenting with refractory FBSS. Of these, six patients (five men and one woman; mean age 67.7±6.4 years; mean BMI 34.9±4.7 kg/m²) underwent a minimally invasive tubular‐assisted approach using the METRx system for the permanent placement of paddle leads (average of 42.5±27.9 days after the initiation of the therapeutic trial). A paramedian incision was made for the surgical approach that was completed with continuous somatosensory evoked potentials (SSEP) and motor evoked potentials (MEP) neuromonitoring. Sequential dilators were docked on the laminar junction at T7-T11 (four at T8-T9, one at T7-T8, and one at T10-T11). After hemilaminectomy and piecemeal flavectomy under direct visualization with microscopic and endoscopic guidance, Boston Scientific paddle leads (Marlborough, Massachusetts, United States) were advanced and confirmed fluoroscopically (AP and lateral) to span the target dorsal columns. Leads were anchored to the interspinous ligament, tunneled subfascially to a gluteal pocket, and connected to an implantable pulse generator.

Results

All paddle leads were successfully placed during the primary procedure. The mean estimated blood loss was 25 mL, and the mean operative time was 56.0±10.6 minutes. There were no perioperative complications, and the average postoperative Visual Analog Scale (VAS) pain score at the first clinical follow-up was 4.5±1.3. Length of follow-up was 2.92±3.13 months. There was no incidence of paddle lead migration, and no replacements or revisions were necessary.

Surgical Technique

**Figure 1:**
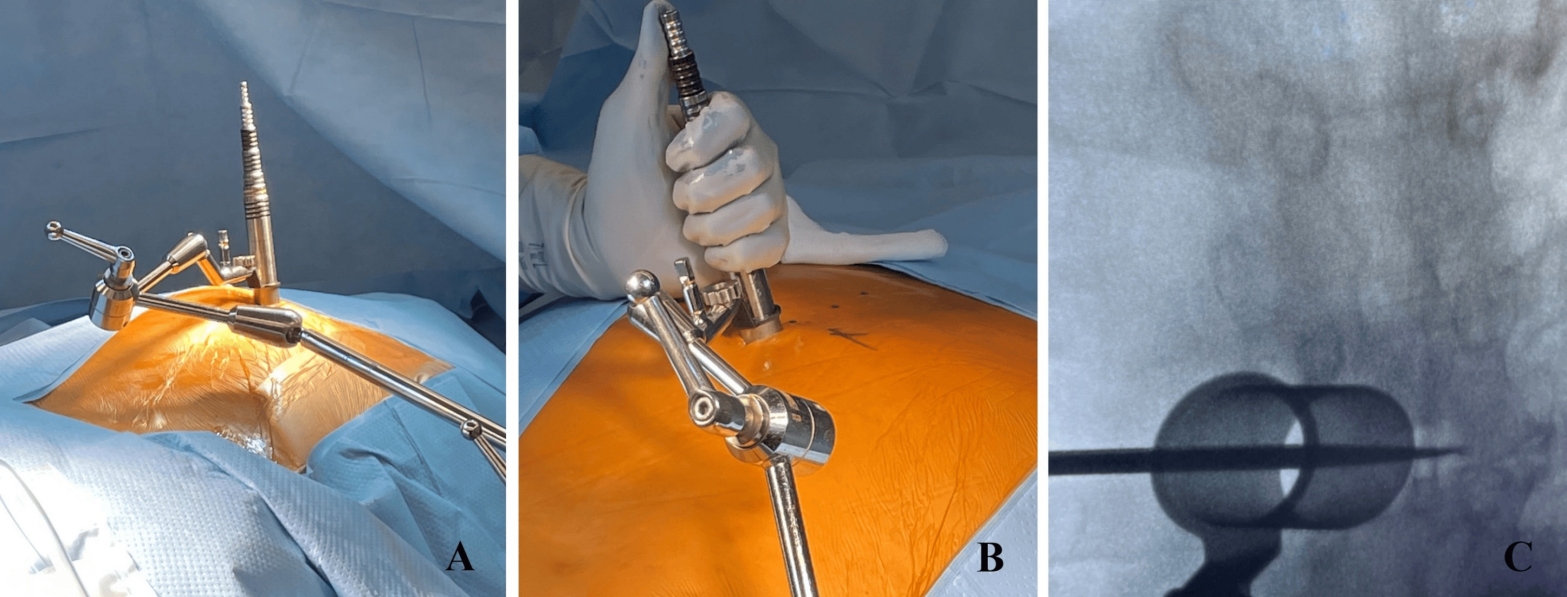
Placement and anchoring of tubular retractors (A) Utilization of tubular dilators for the placement of a paddle-type lead. (B) Demonstration of the final placement of sequential dilators at the level of the thoracic lamina. (C) Verification and anchoring of the tubular retractor spanning appropriate vertebral levels.

**Figure 2:**
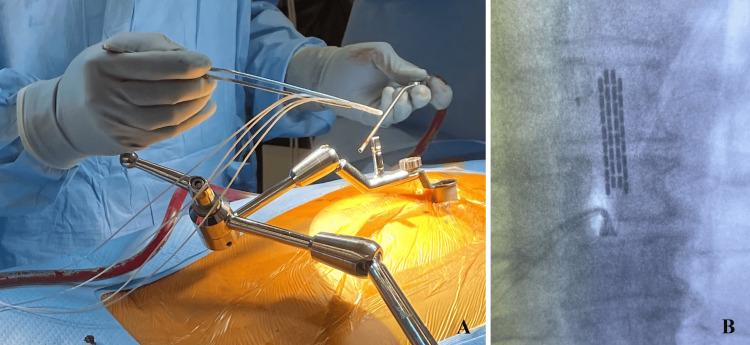
Paddle lead placement (A) Placement of paddle-type lead in the epidural space. (B) Final verification of paddle-type lead placement.

**Figure 3:**
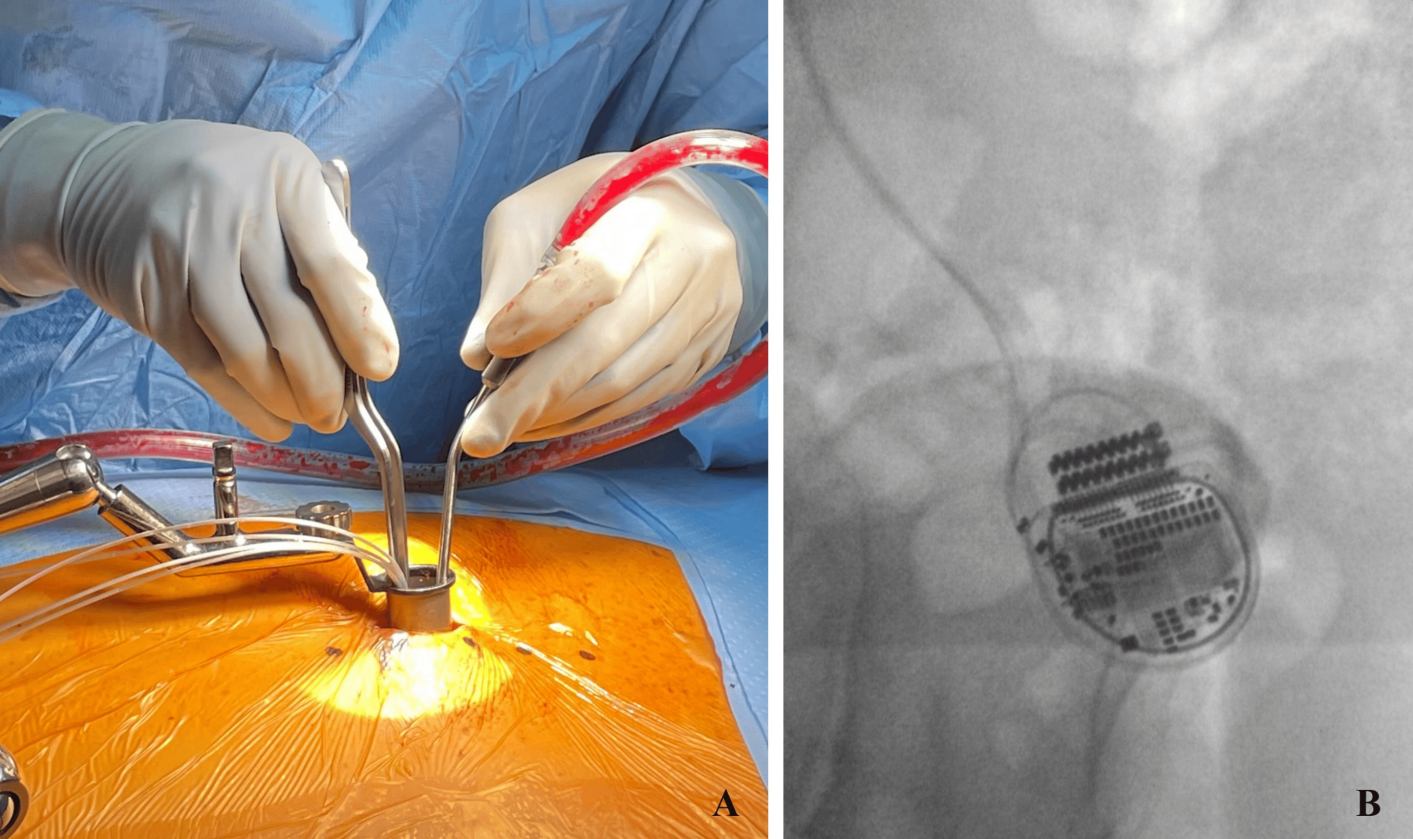
Anchoring paddle and pulse generator (A) Securement of the paddle to the interspinous ligament and (B) subfascial placement of the pulse generator.

After prone positioning on a Jackson table with neuromonitoring (SSEP/MEP) leads placed, intraoperative fluoroscopy was utilized to confirm the appropriate level for a 3 cm paramedian incision. The paraspinal musculature was sequentially dilated utilizing the tubular retractors to create a 22 mm working channel (Figure 1A, 1B). This channel was then docked squarely on the junction of the superior level thoracic lamina and adjacent inferior articular process (Figure 1C). Under direct microscopic or endoscopic visualization, up-biting and straight Kerrison punches were employed to remove the hemilamina of the superior and inferior vertebral lamina. Flavectomy was then performed. The Boston Scientific paddle lead is then advanced cephalad in the exposed dorsal epidural space, with both AP and lateral fluoroscopy used to verify that the paddle spans the appropriate thoracic levels and remains dorsal to the thecal sac (Figure 2A, 2B). Once optimal placement and electrical testing were confirmed, the paddle was anchored to the interspinous ligament to prevent migration (Figure 3A). A subfascial tunnel is fashioned from the thoracolumbar incision to a separate gluteal pocket, where the lead extension is connected to the pulse generator, programmed, and tested before final closure (Figure 3B).

Anatomical Considerations and Technical Pearls

A thorough understanding of the thoracic posterior elements is crucial for safe paddle lead placement. When planning a paramedian approach, the thoracic spinous processes serve as reliable fluoroscopic landmarks for incision planning, while the lamina and inferior articular processes demarcate the ideal docking zone for the tubular retractor. Accurate docking of retractors is necessary not only to facilitate placement of leads but also to prevent unnecessary osteoligamentous disruption of the posterior tension band. Medial dissection into the adjacent facet should be avoided to preserve facet integrity and local spinal stability whenever possible. Once the ligamentum flavum is encountered, piecemeal removal under the microscope exposes the underlying dura without undue traction on the dorsal columns. At this stage, early hemostasis should be ensured to prevent bleeding from the epidural venous plexus at the lamina-pedicle junction that can easily obscure the operative field. Furthermore, extensive adhesions in the epidural space may obscure the dorsal epidural space, preventing accurate paddle positioning. Tubular retractors afford the direct visualization, either endoscopically or microscopically, of this anatomical plane, thereby permitting dissection of such potential adhesions.

## Discussion

While both the METRx tubular approach and a traditional open microdissection achieve safe dorsal column exposure for paddle lead placement, they differ fundamentally in tissue disruption, visualization, and postoperative recovery. The application of sequential dilators and a narrow tubular retractor, often augmented by endoscopic or microscopic assistance, allows for splitting rather than detaching the paraspinal muscles, thereby minimizing blood loss and postoperative pain. Working through a 22 mm channel requires precise fluoroscopic and endoscopic guidance, but it affords excellent midline access with significantly less muscle retraction and fewer fascial incisions. In contrast, the open microdissection approach requires a wider skin incision, subperiosteal muscle reflection off the lamina and spinous process, to achieve direct visualization. While open exposure grants a broader field, potentially easing instrument maneuverability and reducing the learning curve, it disrupts more musculoligamentous tissue, which can translate into increased postoperative pain and longer rehabilitation. Ultimately, while both approaches facilitate the direct visualization of the epidural corridor, the tubular METRx approach offers a minimally invasive corridor with lower physiologic insult, whereas the conventional open microdissection prioritizes maximal exposure at the expense of greater tissue disruption.

Valle-Giler and Sulaiman described a retrospective cohort of 78 patients who underwent MIS placement of paddle lead SCS using a tubular retractor system via a midline approach [17]. This approach minimizes muscle dissection as the tubular retractors were advanced between the rostral and caudal spinous processes through the supra- and interspinous ligaments. In comparison with our proposed paramedian approach, this midline approach affords a reduction in muscular trauma at the expense of increased damage to the posterior tension bands. Valle-Giler and Sulaiman described similar operative outcomes without any major neurologic outcomes [17].

Shamji et al. also investigated the midline approach for MIS paddle lead placement via a prospective, observational cohort study of 20 patients [18]. Ten patients had paddle leads placed in an open fashion, and 10 had paddle leads placed with MIS techniques. When compared to the open approach, the midline MIS approach was associated with shorter operative duration (p=0.03), less blood loss (p<0.001), and less perioperative back pain (p<0.05) [18]. These midline MIS endpoints were comparable to our results and those of Valle-Giler and Sulaiman [17].

Rigoard et al. described a median approach for MIS paddle lead placement in which the interspinous ligament was dissected bilaterally to localize the interspinous space prior to the resection of the supraspinous and interspinous ligaments. This technique was completed on a cohort of 24 patients and achieved comparable clinical and operative endpoints to the aforementioned studies [19].

There have been two other descriptions of the paramedian approach for paddle lead placement via the METRx system by Johnson et al. and Beems et al. [20,21]. Johnson et al. described the minimally invasive technique for implanting multipolar epidural SCS using a tubular retractor system via a paramedial approach in 15 patients. Postoperative complications included one required electrode revision surgery for better placement/successful pain removal and two infections, one of which was at the generator implantation site [20]. Beems et al. also described the utilization of the paramedian approach for the implantation of a paddle lead under local anesthesia. They reported the successful placement of leads in six of the included patients, with one patient failing treatment due to inability to reach the epidural space on account of prior surgery at that level [21].

Our rationale for using a paramedian approach, rather than a midline approach, was to minimize disruption to the posterior tension band. Biomechanical studies have shown that injury to spinal ligaments alters load sharing across adjacent vertebral segments, which may contribute to adjacent segment degeneration [22,23]. Therefore, we reasoned that an approach minimizing such disruption could improve the outcomes of paddle lead placement.

Limitations

In this report, we describe the successful employment of the paramedian approach for paddle lead placement via the METRx system. This report is limited by the small cohort size and follow-up period. Further studies are necessary to determine the efficacy of the paramedian approach to the midline approach for the introduction of paddle leads for SCS.

## Conclusions

Here, we report a novel technique for the placement of paddle-type SCS leads via a paramedian tubular retractor approach in six patients. There was successful lead placement in all cases as well as no perioperative complications. This approach affords a reduction in the disruption of posterior spine ligaments in comparison with previously described midline approaches for the placement of paddle leads via the METRx tubular retractor system. Further prospective studies comparing the midline and paramedian approaches for SCS are necessary to determine the clinical and surgical implications of these approaches.
